# Using the LeiCNS-PK3.0 Physiologically-Based Pharmacokinetic Model to Predict Brain Extracellular Fluid Pharmacokinetics in Mice

**DOI:** 10.1007/s11095-023-03554-5

**Published:** 2023-07-13

**Authors:** Mohammed A. A. Saleh, Berfin Gülave, Olivia Campagne, Clinton F. Stewart, Jeroen Elassaiss-Schaap, Elizabeth C. M. de Lange

**Affiliations:** 1 Division of Systems Pharmacology and Pharmacy, Leiden Academic Center for Drug Research, Leiden University, Gorlaeus laboratorium, Einsteinweg 55, 2333 CC Leiden, The Netherlands; 2 Department of Pharmacy and Pharmaceutical Sciences, St Jude Children’s Research Hospital, Memphis, USA; 3 PD-value B.V., Utrecht, The Netherlands

**Keywords:** brain, leiCNS-PK3.0, mouse, physiologically-based pharmacokinetics (PBPK)

## Abstract

**Introduction:**

The unbound brain extracelullar fluid (brain_ECF_) to plasma steady state partition coefficient, K_p,uu,BBB_, values provide steady-state information on the extent of blood-brain barrier (BBB) transport equilibration, but not on pharmacokinetic (PK) profiles seen by the brain targets. Mouse models are frequently used to study brain PK, but this information cannot directly be used to inform on human brain PK, given the different CNS physiology of mouse and human. Physiologically based PK (PBPK) models are useful to translate PK information across species.

**Aim:**

Use the LeiCNS-PK3.0 PBPK model, to predict brain extracellular fluid PK in mice.

**Methods:**

Information on mouse brain physiology was collected from literature. All available connected data on unbound plasma, brain_ECF_ PK of 10 drugs (cyclophosphamide, quinidine, erlotonib, phenobarbital, colchicine, ribociclib, topotecan, cefradroxil, prexasertib, and methotrexate) from different mouse strains were used. Dosing regimen dependent plasma PK was modelled, and Kpuu,BBB values were estimated, and provided as input into the LeiCNS-PK3.0 model to result in prediction of PK profiles in brain_ECF_.

**Results:**

Overall, the model gave an adequate prediction of the brain_ECF_ PK profile for 7 out of the 10 drugs. For 7 drugs, the predicted versus observed brain_ECF_ data was within two-fold error limit and the other 2 drugs were within five-fold error limit.

**Conclusion:**

The current version of the mouse LeiCNS-PK3.0 model seems to reasonably predict available information on brain_ECF_ from healthy mice for most drugs. This brings the translation between mouse and human brain PK one step further.

## Introduction

Mouse studies recapitulating central nervous system (CNS) diseases have long been used to study human diseases and drug treatment [1, 2], including those related to the CNS [3]. However, such information is not directly translatable to the human situation. Therefore, it is important to seek for translational approaches.

As unbound drug concentration-time profiles (PK) at CNS target sites drive the CNS effect [4, 5] these are most important. Microdialysis is the most adequate technique to assess the unbound drug concentration-time profiles in the brain extracellular fluid (brain_ECF_) and in the different cerebrospinal fluid (CSF) compartments in preclinical species [6–12], but assessment of unbound brain PK in human by microdialysis is highly restricted for ethical reasons. The question is therefore on how to bridge the translational gap between preclinical information to be used in drug development and the clinical setting.

In preclinical species, the unbound brain_ECF_ to plasma steady state partition coefficient, the K_p,uu,BBB_, is a very important value and is often obtained to provide information on the extent of blood-brain barrier (BBB) transport equilibration but is does not provide information on the brain PK profiles, as seen by the brain target sites. It is a ratio, and does not inform on PK profiles, as seen by receptors and other targets.

As PK profiles are driven by the combination of drug properties and the body (system) physiology, the physiological differences between species prevents a direct translation. However, physiologically based pharmacokinetic (PBPK) models explicitly take systems physiology into account, and when combined with drug properties allows prediction of PK profiles, and therefore should be able to bridge the findings between different species, such as rat or mouse and human.

Earlier, we developed a comprehensive CNS PBPK model in rat and human, the LeiCNS-PK3.0 [13, 14]. This model includes the BBB and blood-CSF barrier (BCSFB) characteristics and surfaces, the brain_ECF_, brain intracellular fluid (brain_ICF_), cerebrospinal fluid (CSF) in lateral ventricles, third and fourth ventricles, cisterna magna, and subarachnoid space, and their volumes and flows, and the brain cell membranes surfaces, brain cell volumes, lysosome volumes, and pH values in all the compartments. With that it allows prediction of a drug’s blood to brain and intra-brain transport processes, including nonspecific binding, when the drug properties were used as input together with the plasma PK after the mode of drug administration of choice. The predicted observed unbound drug PK in brain_ECF_ and different CSF compartments in rats and humans were within less than two-fold error, demonstrating rat-to-human translatability of CNS PK profiles [13].

A LeiCNS-PK3.0 mouse version could help to translate mouse CNS PK to that of human, and thereby bridge a lot of mouse data for human interpretation. In this study, we searched for healthy mouse CNS physiological parameters from literature, and connected unbound plasma PK, and associated brain_ECF_ PK, as obtained by microdialysis. Such data was available for 10 drugs, from different mouse strains/types, with different physicochemical properties. Using the plasma data, plasma PK models were developed, to inform the LeiCNS-PK3.0 model, together with the detailed mouse CNS physiological parameters and calculation of K_p,uu,BBB_. Here we report and discuss the results on the performance of this model to predict brain_ECF_ data in mice.

## Data and Methods

The LeiCNS-PK3.0 model structure [13] was informed on mouse CNS physiological information and mouse drug unbound plasma and associated brain_ECF_ PK as far as available from literature. For 10 drugs such PK information was available, and plasma PK model were available for 3 drugs and for 7 drugs a plasma PK model was developed. Furthermore, the physicochemical and biological properties of these drugs were obtained/ calculated. All is explained below.

### Drugs

The following 10 drugs were used to evaluate the predictive performance of the mouse version of the LeiCNS-PK3.0 model: cyclophosphamide, quinidine, erlotonib, phenobarbital, colchicine, ribociclib, topotecan, cefradroxil, prexasertib, and methotrexate.

### Drug physicochemical properties

The physicochemical properties of the drugs were extracted from DrugBank release version 5.1.9 [15] and are presented in Table I. Lipophilicity (as logP) was estimated using the ALOGPS [16], while the acid/base ionization constants, polar surface area, and hydrogen bond donor/acceptor values were provided by the Chemaxon method [17].

**Table I:** Physicochemical Properties of the 10 Drugs

	Mwt (g/mol)	logp	pka	pkb	HBA	HBD	PSA (Å^2^)
cyclophosphamide	261	0,76	13,48	NA	1	1	41,57
quinidine	324	2,82	13,89	9,05	4	1	45,59
erlotinib	393	3,13	16,14	4,62	7	1	74,73
phenobarbital	232	1,4	7,14	NA	3	2	75,27
colchicine	399	1,59	15,06	-1,2	6	1	83,09
ribociclib	435	2,5	11,59	8,87	7	2	91,21
topotecan	421	1,84	8	9,75	6	2	103,2
cefadroxil	363	0,51	3,25	7,22	6	4	132,96
prexasertib	365	1,77	10,02	9,85	8	3	134,76
methotrexate	454	-0,91	3,41	2,81	12	6	205,92

### *In Vivo* Data

Plasma and associated brain_ECF_ concentration-time profiles (Table II) of part of the drugs were kindly provided by the Stewart lab (St. Jude Children's Research Hospital, Memphis, Tennessee, USA) or otherwise extracted from literature with WebPlotDigitizer version 4.5 (https://automeris.io/WebPlotDigitizer/).

**Table II:** Sources of Mouse Plasma (with fu,plasma) and Associated brainECF PK Data

	Mouse strain/type	Plasma	Brain_ECF_	fu,plasma	Reference data	Refs plasma PK model
cyclophosphamide	CD1 nude	X	X	0,26	St Judes	[7]
quinidine	NMRI	X	X	0,233	[18]	in-house NONMEM
erlotinib	FVB	X	X	0,048	St Judes	in-house NONMEM
phenobarbital	ICR	X	X	0,7	[19]	in-house NONMEM
colchicine	NMRI	X	X	0,61	[20]	in-house NONMEM
ribociclib	CD1 nude		X	0,23	[9]	[9]
topotecan	CD1 nude	X	X	0,3	St Judes	in-house NONMEM
cefadroxil	C57BL/6/Pept2+/+	X	X	1	[21]	in-house Monolix
prexasertib	CD1 nude	X	X	0,11	[22]	[22]
methotrexate	CD1 nude	X	X	0,519	St Judes	in-house NONMEM

^a^Data were kindly provided by Prof. Dr. C F Stewart from St. Jude Children's Research Hospital, Memphis, Tennessee, USA.

### Mouse CNS Physiological Parameters

Mouse parameter values for CNS physiology were obtained from literature. Values for BBB and blood-CSF barrier (BCSFB) characteristics and surfaces; brain_ECF_, brain_ICF_, CSF in lateral ventricles, third and fourth ventricles, cisterna magma and subarachnoid space, volumes and flows; brain cell membranes surfaces, brain cell volumes, lysosome volumes; and pH values in all the compartments were collected. In case multiple values were found, the mean value was calculated. The surface area of the BBB was calculated with two approaches and the mean value was computed. The first, using the microvessels average radius, length density, and brain volume, while the second using the brain vessel surface area to brain volume ratio and total brain volume.

### Plasma PK Modeling

The plasma PK models of cyclophosphamide, ribociclib, and prexasertib were available from literature [7, 9, 22]. The plasma PK parameters of cefadroxil were estimated using Monolix version 2021R2 (Lixoft, Orsay, France). Plasma PK model parameters of the other 6 drugs were estimated using NONMEM version 7.4.3 (ICON, Dublin, Ireland) [23]. Population plasma PK models were developed and used as input to the CNS PBPK model. In brief, one-, two-, three- compartment models were fitted to total plasma concentrations, accounting for the associated interindividual variabilities (where possible) using an exponential function, and for the residual unexplained variability using proportional or combined proportional and additive error models. The final model was selected based on likelihood ratio test with p<0.05 corresponding to an objective function value decline of 3.84, visual predictive check (VPC) plots, precision of the parameter estimates (%RSE), and the basic goodness of fit plots.

### Drug Biological Properties–Calculation of K_p,uu,BBB_ Values

K_p,uu,BBB_ values, defined as the ratio of the unbound drug in brain_ECF_ to that of plasma at steady state, reflect the extent of drug transport across a barrier (i.e. BBB or BCSFB). These values may differ from 1 due to transporters at these barriers [24]. K_p,uu,BBB_ values were calculated by the ratio of influx and efflux clearances across the BBB, respectively, or by the ratio of the AUC 0-∞ at the brain_ECF_ to that of plasma, respectively [24]. Where unavailable, the influx and efflux clearances of the unbound drug across the BBB were estimated by combining the respective population plasma PK model and a one-compartment model representing the whole brain. Then, these K_p,uu,BBB_ values were used to calculate the asymmetry factors at the BBB that reflect the net active transport across these barriers in the LeiCNS-PK3.0 model.

### Mouse LeiCNS-PK3.0 Model Evaluation and Data Analysis

As indicated, the LeiCNS-PK 3.0 mouse model was developed using the previously published model structure of the rat and human LeiCNS-PK3.0 model versions. The mouse CNS physiological parameters were given as input, together with the K_p,uu,BBB_ values, and the plasma PK parameters. The LeiCNS-PK3.0 mouse model predictions of brain_ECF_ were evaluated by comparison with the observed CNS PK data, using visual predictive checks (VPC). In addition, using the prediction errors, the percentage average fold error (%AFE) and percentage absolute average fold error (%AAFE) were computed as described previously [13] and were used to evaluate the bias and the accuracy of the model predictions, respectively. Data analysis and visualization were performed in R (version 4.1.2) [25]. The LeiCNS-PK3.0 model simulations were also performed in R, using the package RxODE (version 1.1.4) and the LSODA (Livermore Solver for Ordinary Differential Equations) Fortran package [26].

## Results

### Mouse CNS Physiological Parameters

Mouse parameter values for CNS physiology were obtained from literature. When more values of a certain parameter were found, the mean value was used in the LeiCNSPK3.0 mouse model. Table III lists all parameter values and relevant assumptions.

**Table III:** Collected Values of Mouse CNS Physiological Parameters

Aspect (units)	parameter	value	final value	Reference
Volume (μl)	Total brain	303	360	[27]
		360		[28]
		495		[29]
		150		[30]
		350		[31]
		360		[32]
		360		[33]
	Brain_ECF_	67	67	[28]
	Brain_ICF_		288^a^	[13]
	Total lysosome		3.6^b^	[13]
	Total CSF volume		35	[34]
	Total ventricles		4.8	[35]
	Lateral ventricles	4	1.0275	[36]
		0.4		[31]
		2		[30]
		0.79		[37]
		0.32		[37]
		0.46		[37]
		0.12		[37]
		0.13		[37]
	3^rd^ & 4^th^ Ventricles		2.5	[36]
	Cisterna magna		2.13^c^	
	Subarachnoid space		16.88^c^	
	Microvasculature		5	[28]
Flow (ml/min)	Cerebral blood flow	0.46134	0.46134	[33]
		0.46134		[32]
	Brain_ECF_ flow	0.0001248	0.0003744^d^	[38]
		0.000624		[38]
	CSF flow	0.000325	0.000343	[39]
		0.000361^e^		[40]
Surface area (cm^2^)	BBB	18.78	19.76	[35, 41]
		20.74		[35, 42]
	BCSFB	9.88	9.88^f^	
	BCM	1006.5	1006.5^g^	
	Lysosomes	540	540^h^	
Effective SA (unitless)	BBB-transcellular		0.998^i^	[43]
	BCSFB-transcellular		0.998^i^	[43]
	BBB-paracellular		0.006^i^	[43]
	BCSFB-paracellular		0.05^i^	[43]
Width (μm)	BBB		0.7	[44]
	BCSFB		1.7	[44]
Volume fraction (unitless)	Brain phospholipids		0.05	[45]
pH (unitless)	Plasma		7.4	[46]
	Brain_ECF_		7.4	[47]
	Brain_ICF_		7.2	[47]
	Lysosomes	4.8^j^	5.5	[48]
		6^k^		[49]
		5.5^l^		[50]
		5.6		[51]
		4.9^j^		[52]
	CSF		7.2	[53]
Brain (count)	Brain cell number		108,690,000	[54]

^a^80% of total brain volume (median total brain volume) [13]

^b^1.25% of brain intracellular fluid volume [13]

^c^Assuming equal ratio of total cerebrospinal fluid (CSF) and cisterna magna/subarachnoid space in rats and mice

^d^based on a mouse brain weight of 0.416 g

^e^CSF flow = 40/1000 (CSF volume ml) * 13 (CSF turnover /day)/(24*60)

^f^assuming BCSFB = 50% BBB [55]

^g^based on brain cell number and ICF volume

^h^based on lysosomal radius and lysosome volume

^i^assumed the same in rodents [43]

^j^From neurons

^k^From microglia

^l^From astrocytes

### Plasma PK Modelling

**Fig. 1:**
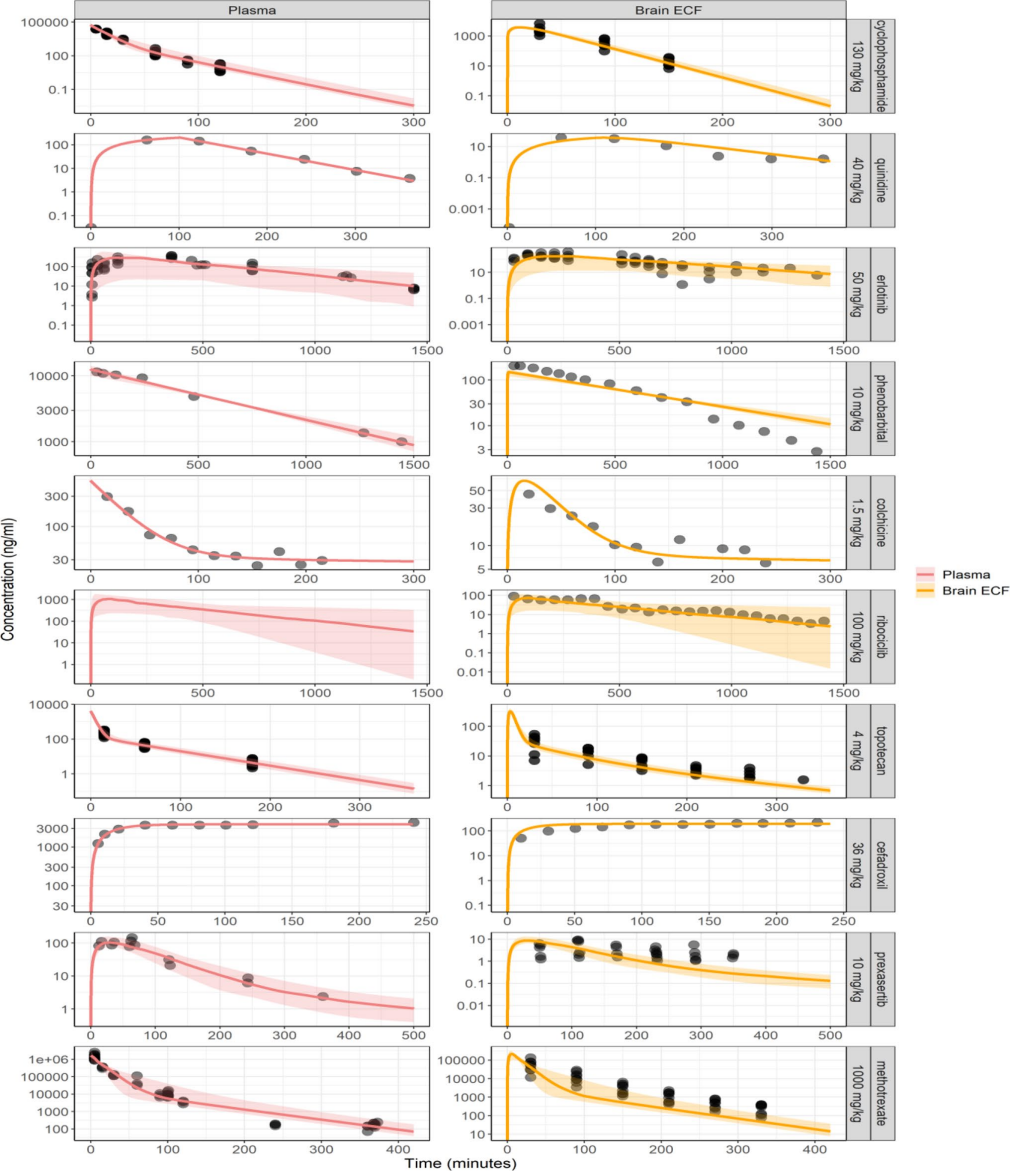
**Visual predictive check plots evaluating the predictive accuracy of the mouse version of the LeiCNS-PK3.0 model.** Ten drugs with different physicochemical properties and affinities to active transporters were used to evaluate the model predictions. The solid lines and colored band represent the median and 95% prediction interval, respectively, of the model’s prediction of the unbound pharmacokinetic profile at the plasma (red), brain extracellular fluid (yellow). The black dots represent the unbound drug concentrations measured in mice. Drugs were simulated with various routes of administrations: cyclophosphamide, quinidine and phenobarbital were intraperitoneal; erlotinib and ribociclib were orally; prexasertib was subcutaneous; colchicine, topotecan, cefadroxil and methotrexate were intravenously administered. Please note the different axes scales

**Fig. 2:**
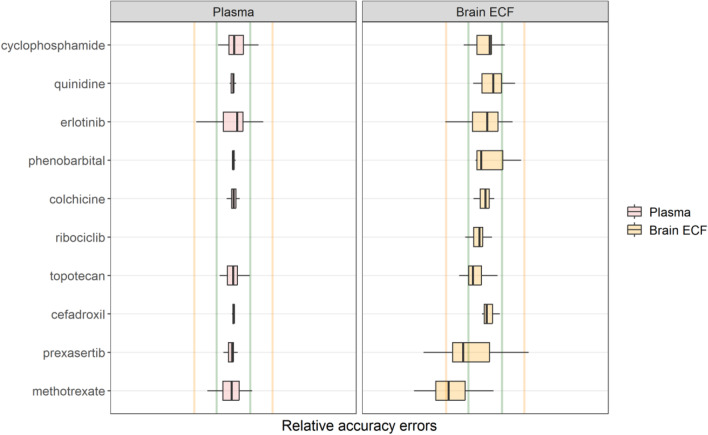
**Box plot of the relative accuracy errors to evaluate the prediction accuracy of the current mouse version of the LeiCNS-PK3.0 model.** The predictions of the ten drugs in plasma (red) and brain extracellular fluid (yellow) were evaluated using the relative accuracy errors. The green and yellow vertical lines represent two- and five-fold error limit, respectively. The predictions of methotrexate and prexasertib were beyond the two-fold errors but were within the five-fold error

For a given dose regimen, for the LeiCNS-PK3.0 model the associated plasma PK model parameters were used as input and forcing function as an input and forcing function to reduce errors due to the potential imprecise plasma PK predictions of a whole body PBPK model. The plasma PK models parameters are reported in Table IV and the model prediction of brain_ECF_ data against the observed brain_ECF_ data and associated errors are depicted in Figs. 1 and 2, respectively. Generally, the models were estimated within two-fold error and the *in vivo* plasma PK data were accurately described.

**Table IV:** Plasma PK Parameters of the Different Drugs

	cyclophosphamide	quinidine	erlotinib	phenobarbital	colchicine	ribociclib	topotecan	cefadroxil	prexasertib	methotrexate
Dose (mg/kg)	130	40	50	10	1.5	100	4	36	10	1000
Route of administration^a^	IP	IP	PO	IP	IV	PO	IV	IV	SC	IV
Empirical plasma PK models										
fu, plasma	0,26	0,233	0,048	0,7	0,61	0,23	0,3	1	0,11	0,519
Central clearance (ml/min)	2.09	46.99	0.412	0.02	0.487	1.32	1.41	0.97	2.68	0.688
Intercompartmental clearance (ml/min)	0.086	0	0	0	2.11	0	0.486	0	0.276	0.03
Central compartment volume (ml)	18.53	2951.3	147	11.2	70.1	530.1	8.41	14	51.59	9.21
Peripheral compartment volume (ml)	1.77	0	0	0	705	0	18.36	0	97.47	2.14
1^st^ order absorption (1/min)	0	2.61	0.012	0	0	0.046	0	0	0.018	0
Duration (min)	0	101	0	0	0	0	0	0	0	0
Interindividual variability (as variance)										
Central clearance	0.012	0	0	0	0	0.217	0.011	0	0.046	0.021
Intercompartmental clearance	0	0	0	0	0	0	0	0	0.027	0
Central compartment volume	0	0	0	0	0	0	0	0	0	0
Peripheral compartment volume	0	0	0	0	0	0	0	0	0	0
1^st^ order absorption	0	0	0.614	0	0	0.676	0	0	0.04	0
Residual unexplained variabilities (as variance)										
Proportional error	0.058	0	0.212	0.005	0.0224	0.276	0.051	0	0.003	0.18
Additive error (ng/ml)	0	2.05	0	0	0	0	0	0	0	0

^a^Routes of administration: IV = intravenous and IP = intraperitoneal, PO = oral, SC = subcutaneous

### Drug Biological Properties – Calculation Of K_p,uu,BBB_ Values

As input for the LeiCNS-PK3.0 model values of the asymmetry factors, the K_p,uu,BBB_ values for the different drugs were calculated as clearance in over clearance out of the brain_ECF_. Results are shown in Table V.

**Table V:** Kp,uu,BBB Values for the 10 Drugs

	Kp,uu,BBB
cyclophosphamide	0.339 (estimated)
quinidine	0.2185 [56]
erlotinib	0.628 (estimated)
phenobarbital	0.0121 (estimated)
colchicine	0.14 [20]
ribociclib	0.0693 [9]
topotecan	0.21 [57]
cefadroxil	0.05 (estimated)
prexasertib	0.09 [22]
methotrexate	0.195 (estimated)

### Mouse LeiCNS-PK3.0 Model Evaluation And Data Analysis

Model validation was performed by comparing the data-independent LeiCNS-PK3.0 model predictions at brain_ECF_ to drug concentrations measured *in vivo* with microdialysis (Fig. 1). Overall, the LeiCNS-PK3.0 model predictions were good for 7 out of 10 drugs. For phenobarbital, the prediction of brain_ECF_ was slower (lower Cmax, slower elimination rate) than the actual data. For prexasertib, the model predictions of brain_ECF_ were faster (higher and earlier Cmax, higher elimination rate), while for methotrexate the model prediction of underestimated the elimination phase of brain_ECF_ data.

The LeiCNS-PK3.0 model bias was assessed using relative accuracy errors (%AFE), which was 99.6% and 76.5%, for plasma and brain_ECF_, respectively. The model’s predictivity of the typical CNS PK profile was evaluated using the %AAFE, which was 105 % and 152 %, for plasma and brain_ECF_, respectively. Figure 2 displays the visual predictive checks. It shows that the LeiCNS-PK3.0 mouse model could adequately predict the brain_ECF_ drug concentrations, within the two-fold error limit for 8 out of the 10 drugs.

## Discussion

Translation between mouse and human CNS PK data would be an important step forward in CNS drug development, as animal data can be used in a better way. Lots of total plasma and total brain concentrations in mice are available, however, the link to the human situation cannot be directly made. The K_p,uu,BBB_ (or K_p,uu,ECF_) value can be obtained using steady-state ratios of unbound brain over unbound plasma concentrations, but this ratio can have the same value for multiple combinations of plasma and brain_ECF_ PK. Brain targets, mostly extracellular, however, see the PK profiles, and therefore we need insights into the relationship between plasma PK and brain_ECF_ PK profiles. In this study we explored the use of the LeiCNS-PK3.0 model to predict brain_ECF_ PK profiles, based on unbound plasma PK profiles. If such a model would be adequate, it may be used to be further extended to other CNS compartments, and ultimately may also use mouse K_p,uu,brain_ values to predict brain_ECF_ and other CNS location PK profiles for translation to the human situation.

In this study, we validated the LeiCNS-PK3.0 model for its use to predict mouse brain_ECF_ data. Earlier versions of the LeiCNS-PK3.0 model have shown to adequately predict rat and human CNS unbound PK profiles in multiple CNS physiological compartments [13]. Here we used all available data on unbound plasma PK and associated brain_ECF_ PK profiles, as well as literature information on details of the mouse CNS physiology, to explore the ability of this mouse version of the LeiCNS-PK3.0 model to predict brain_ECF_ data.

Many published studies have reported the development of whole-body mouse PBPK models, accounting also for the brain [10, 32, 58–62]. These models were used to predict mouse PK profiles in multiple organs and to translate the PK profiles to humans. However, these models do not distinguish the brain cells, brain_ICF_ and brain_ECF_ [32, 58], while also do not account for the presence of lysosomes and non-specific binding [10, 32, 58]. Explicit distinction between all CNS physiological compartments, particularly the main target sites: brain_ECF_ and brain_ICF_, is very relevant for more accurate assessment of the concentration-effect relationship [63]. Our LeiCNS-PK3.0 model inputs are physiological parameters, drug physicochemical properties, and K_p,uu_ values, which can be obtained from *in vivo*, *in vitro* [64], or in silico [65] studies. None of the model parameters was estimated and, therefore, the model is translatable to other species, including humans, and to predict the CNS PK of small molecule drugs.

The current mouse LeiCNS-PK3.0 model is the first mouse CNS PBPK model of small molecule drugs, to the best of the authors’ knowledge, that accounts mechanistically for the mouse CNS physiology, including the different compartments and drug transport modes, bulk fluid flow, pH differences, and non-specific binding. The first step was to see if the model could adequately predict brain_ECF_, of available mouse data sets with associated plasma PK profiles, with fu,plasma information, and brain_ECF_. 10 drugs were found (cyclophosphamide, quinidine, erlotonib, phenobarbital, colchicine, ribociclib, topotecan, cefradroxil, prexasertib, and methotrexate), with data from different mouse strains/types. Overall, the LeiCNS-PK3.0 model brain_ECF_ predictions were good for 7 out of 10 drugs. For phenobarbital, the prediction of brain_ECF_ was slower (lower Cmax, slower elimination rate) than the actual data. For prexasertib, the model predictions of brain_ECF_ were faster (higher and earlier Cmax, higher elimination rate), while for methotrexate the model prediction might overestimated the Cmax and underestimated Tmax (not enough early time data to know), while it underestimated the elimination phase of brain_ECF_ data. This could not be due to the plasma PK input, as all models rather precisely described the plasma PK profiles. Analytical assays might also be a source of some deviation, but not detailed enough information on the analytical assays for high and low concentration CV% were provided to assess this possibility. Potential differences between the CNS physiology of the mouse types/strains could contribute, but the number of drugs studied with the unbound plasma and brain_ECF_ data is too little to further analyze such a possibility. More data should be produced to further evaluate the mouse LeiCNS-PK3.0 model, while its performance is already quite a step forward.

Another aspect could be the mouse BBB surface area (SA). It is reported in literature to be 240 cm^2^/g brain equivalent to 86.4 cm^2^ for a 360-μg mouse brain [66]. This value when used in the LeiCNS-PK3.0 model resulted in poor prediction of brain_ECF_ PK profile (results not shown). In comparison, BBB SA in rats and humans were 82 and 120 cm^2^/g brain, respectively [13], implying that 240 cm^2^/g brain could be an overestimation of mouse BBB surface area. Hence, we calculated a mean mouse BBB SA of 19.8 cm^2^ using two techniques: a value of 18.8 cm^2^ using the surface area per unit volume of different brain regions [42], weighted by the regional volume [35] and corrected for the total brain volume and another value of 20.7 cm^2^ using the average microvessels diameter and length density, corrected for total brain volume [41, 42]. The new value resulted in better prediction of brain_ECF_ PK profiles of cyclophosphamide and topotecan, while that of other drugs in our dataset remained the same. This approach is what we call the “handshake approach” [67], as in our opinion, we can especially learn back from *in vivo* data, and therefore (CNS) PBPK models should not only be informed by *in vitro* or *in silico* information, to improve physiological parameter values in the PBPK models.

Besides methodological and/or physiological aspects, drugs physicochemical properties could play a role in passive BBB transport. We considered the polar surface area (PSA) to play a role [68], being relatively high for methotrexate and prexasertib (206 and 135 Å^2^, respectively). However, cefadroxil also has a high PSA value (133 Å^2^), but could be predicted within two-fold error, while that of phenobarbital is much lower (75 Å^2^) and not within two-fold error. Another consideration was to compare the number of hydrogen bond acceptors (HBA) and/or donors (HBD). For methotrexate HBA/HBD was 12/6, and for prexasertib it was 8/3. However, for phenobarbital this was 3/2. So, no clear pattern for HBA and HBD neither. Then, the comparison of the mouse *versus* the rat CNS LeiCNS-PK3.0 model performance could only be done for methotrexate, as the only drug for which appropriate data was observed in mouse and rat. In the rat model, methotrexate brain_ECF_ data were within 251 %AAFE *versus* 433 %AAFE for that in mice. Altogether, this indicates the need for more in-depth analysis of the combination of multiple physicochemical properties, as well as exploring potential physiological aspects that may vary between different mouse strains and/or methodologies used to measure the physiology. Although our goal is to reuse as much animal data as possible, and to save animal lives, It might even be necessary to have additional microdialysis data on CNS drug distribution in mice produced, in which also other CNS locations and end-of-experiment total brain concentrations can be obtained in conjunction (connected data, [69].

This model will be further improved in depth analysis of the influence of drug physicochemical properties. Furthermore, using the “handshake” approach [67], the impact of physiological values used in the model will be studied, and improved, assuming that *in vivo* data “tell the truth”. Next steps will be to make use of *ex-vivo* plasma, and plasma binding, as well as brain homogenate and brain binding, to calculate K_p,uu,brain_ values [70], by which the model can predict full pharmacokinetic profiles in the different compartments”.

Altogether, the current mouse LeiCNS-PK3.0 model shows adequate predictions of observed brain_ECF_ data for 7 out of the 10 drugs for which the unbound plasma PK and associated brain_ECF_ data were available. While some deviating predictions were also observed, the mouse LeiCNS-PK3.0 holds promise for further development to be useful as a translational tool to predict the healthy, and ultimate diseased human CNS PK profiles, also from using PK data obtained from mice.

## Data Availability

Data sharing is not applicable to this article as no new data was generated in this study.
